# Polyphenolic Spectrum of Goji Berries and Their Health-Promoting Activity

**DOI:** 10.3390/foods14081387

**Published:** 2025-04-17

**Authors:** Tunde Jurikova, Simona Morvay Tinakova, Jana Ziarovska, Ladislav Szekeres, Jiri Mlcek, Katarina Fatrcova-Sramkova, Zuzana Knazicka, Sona Skrovankova

**Affiliations:** 1 Institute for Teacher Training, Faculty of Central European Studies, Constantine the Philosopher University in Nitra, Drazovska 4, 949 01 Nitra, Slovakia; tjurikova@ukf.sk (T.J.); lszekeres@ukf.sk (L.S.); 2 Medic Cells s.r.o., 949 01 Nitra, Slovakia; tinakova@mediccells.sk; 3 Department of Plant and Environmental Sciences, Faculty of Agrobiology and Food Resources, Slovak University of Agriculture, Trieda Andreja Hlinku 2, 949 76 Nitra, Slovakia; jana.ziarovska@uniag.sk; 4 Department of Food Analysis and Chemistry, Faculty of Technology, Tomas Bata University in Zlín, Vavreckova 5669, 760 01 Zlín, Czech Republic; skrovankova@utb.cz; 5 Institute of Nutrition and Genomics, Faculty of Agrobiology and Food Resources, Slovak University of Agriculture, Trieda Andreja Hlinku 2, 949 76 Nitra, Slovakia; katarina.sramkova@uniag.sk (K.F.-S.); zuzana.knazicka@uniag.sk (Z.K.)

**Keywords:** goji berry, *Lycium* spp., polyphenol, anthocyanin, antioxidant activity, health benefits

## Abstract

A significant increase in interest in new, naturally occurring sources of antioxidants is evident not only in the food industry but also in the pharmaceutical and cosmetic industries. Plant sources such as fruits, both traditional and less common, are often investigated. Goji berries (*Lycium barbarum*, *Lycium chinense*, and *Lycium ruthenicum*) represent fruits rich in polyphenols, especially phenolic acids (38.91 to 455.57 mg/kg FW) and flavonoids, with black goji berries (*L. ruthenicum*) containing a predominance of anthocyanins (119.60 to 1112.25 mg/kg FW). In this review, a comparison of polyphenol occurrence and content in the orange-red and black berries of *L. barbarum*, *L. chinense,* and *L. ruthenicum* is described. Goji berries represent a valuable source of nutrients and bioactive compounds that manifest a wide range of health-promoting effects. These benefits represent antioxidant, neuroprotective, and cytoprotective impacts, with effects on the metabolic control of glucose and lipids. This review is focused on an overview of the polyphenolic compounds occurring in these fruits, as well as their antioxidant activity and health benefits.

## 1. Introduction

Goji berries of the *Lycium* genus belong to the Solanaceae family. The genus includes closely related species that are the most cultivated and utilized: barbary wolfberry (*Lycium barbarum* L.) and Chinese boxthorn (*Lycium chinense* Mill.). They have ellipsoid orange-red berries that are known for their sweet, tangy flavor. The goji fruits of *Lycium ruthenicum* Murr. have a sweet, tangy, and pungent taste and black berries, and are less utilized than the other mentioned species, even though they have important edible value and medicinal qualities [1,2].

Goji berries are widely distributed in China, especially in its northwestern part, such as the Ningxia region and the provinces of Gansu, Xinjiang, and Qinghai, as well as in North and South America and Western Europe, where the fruit was introduced in the 18th century [3]. Commercial goji berries are mainly produced in the above-mentioned areas of China, Mongolia, Korea, and Japan. In 2022, the harvesting area of goji berry reached 62,600 acres, and the total fresh fruit output was 300,000 tons, with its processing conversion rate being 30% solely in Ningxia, a Northwestern Chinese autonomous region [4].

Goji berries represent a great source of nutrients and bioactive compounds, such as lipids, with the neutral lipid contents of the commercially available fruit of *L. barbarum* and wild *L. ruthenicum* being 6.1 and 7.5%, respectively. They also contain proteins—with the most abundant essential amino acids being proline and serine—polysaccharides (comprising 5–8% of the dried fruits), dietary fiber [1,5,6,7,8], vitamin C [6,9,10], and minerals, especially potassium, copper, manganese, iron, and zinc [10,11]. The important biologically active compounds, which include phenolic compounds, carotenoids, phenylpropanoids, coumarins, lignans, and their derivatives, are of great significance as well. From the phenolics, the most valued are phenolic acids and flavonoids [1,11,12,13,14,15].

**Figure 1:**
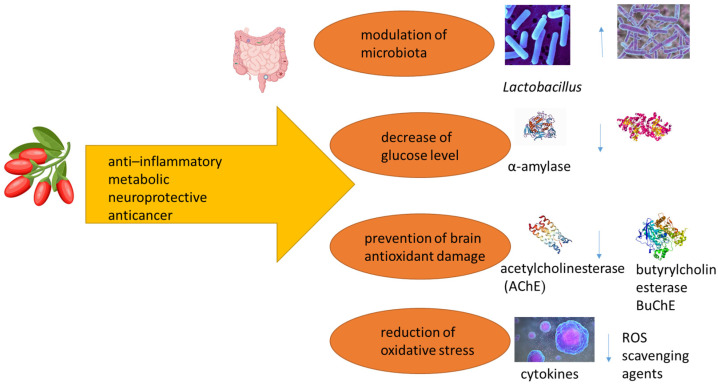
Prophylaxis of polyphenols in goji berries.

Goji fruit is also gaining attention as one of the most modern functional foods due to the use of its juice, peel, and seeds [1,4], with a wide range of supporting health activities of the available substances, supplementing the Western diet [16]. It is even considered a superfood in Europe and North America [3]. The prophylaxis effect is illustrated in Figure 1.

Worldwide, three general strategies to process and utilize goji berry plants have been distinguished: (1) the primary processing of goji berry products (dried goji berry pulp and fruit wine with its by-products); (2) the deep processing of sugar peptides and carotenoids; and (3) the extraction and utilization of plant-based by-products (roots, stems, leaves, flowers, and fruit residuals) [2].

The fruit, stems, leaves, and roots are used in traditional Chinese medicine [17] in the treatment of diabetes, hyperlipidemia, hepatitis, immune disorders, thrombosis, male infertility, and cancer [18]. Goji berries’ health-promoting activities can be characterized by the combined effect of their individual components, nutrients, and bioactive components [1,14]. The berries are commonly consumed in fresh form, dried, or processed. They can be used in berry infusions, juice, wine, liqueur, jams, sauces, salads, beer, or additives (e.g., to ice cream and baked and dairy products) [1,19,20,21].

The goji fruit displays a wide range of health-promoting effects such as anti-aging, neuroprotective, cytoprotective, antioxidant, and immunomodulatory impacts. It is effective in the metabolic control of glucose in diabetics and in lipid reduction [19,22,23].

Many research studies have focused on the health properties of polysaccharides and carotenoids in goji berries. On the other hand, polyphenols are also an important group of compounds that contribute to the high antioxidant activity and health benefits of this fruit. Therefore, this review summarizes and discusses the individual polyphenolic substances, their content in different species (*Lycium barbarum*, *Lycium chinense* and *Lycium ruthenicum*), and their health benefits from different perspectives.

## 2. Total Polyphenolic Content (TPC) of Goji Berries and Factors Influencing Polyphenol Content

Goji berries are a rich source of polyphenols, one of the primary groups of bioactive compounds with a wide spectrum of biological activities.

Ağagündüz et al. [24] determined the total polyphenol content of dry goji berry fruits (*L. barbarum*) harvested in Turkey. The TPC was determined by the Folin–Ciocalteu method, with an average value of 207.2 mg GAE (gallic acid equivalents)/100 g FW (fresh weight). Ilić et al. [25] studied the total polyphenol content of methanolic extract of *L. ruthenicum* berries from Serbia using the Folin–Ciocalteu microplate method. They found a TPC value of 1459 mg GAE/100 g DW (dry weight) in the goji sample.

The differences among goji species are also noticeable in the study of Kafkaletou et al. [26], who studied six genotypes of *L. barbarum* and one genotype of *L. chinense*, derived from dried fruits obtained from different retail markets in Europe and originating from China. *L. chinense* cultivated in Greece accumulated higher levels of phenolics (4–13 mg/g DW) and other antioxidants in its organs than *L. barbarum*. By attenuated total reflection FTIR (ATR Fourier-transform infrared spectroscopy), Skenderidis et al. [27] provided a comparative analysis of the phenolic content of goji fruits originating from two species (*L. barbarum* and *L. chinense*) from plantations located in Greece, one *L. barbarum* from Mongolia, and another from China. The results of their study showed that the fruits of Greek *L. barbarum* presented higher concentrations of phenolics (6.9–10.1 mg GAΕ/g) than *L. chinense* Mill. (7.4–8.9 mg GAΕ/g). Furthermore, the TPC value from the Chinese fruit was lower (9.9 mg GAE/g) than for the Mongolian fruit (10.9 mg GAE/g). Species variance is closely linked with the color of the fruit. Significant differences were noticed among black, red, and yellow goji berries. The experiments showed higher total phenolic content (8.33 mg GAE/g) in black goji berry samples compared to red goji berry (3.16 mg GAE/g) [28]. A comparative study [29] of black and red goji berries showed that black goji berry samples had a relatively higher total phenolic content (9.01, 8.95, 8.08, and 7.26 mg GAE/g DW) compared to red goji berry samples (2.17, 2.87, 3.12, and 4.48 mg GAE/g DW). Ilić et al. [11] studied and compared the bioactive compounds of three different samples of *L. barbarum* L., red and yellow, and black goji berry (*L. ruthenicum* Murr.) cultivated in Serbia. The results proved that the highest level of TPC and the highest antioxidant activity were observed for the extract of black goji berry.

Another important factor is the place of cultivation. The origin of samples and its effect on total polyphenol content was evident in the study by Rajkowska et al. [30]. They compared the phenolic content of naturally dried and freeze-dried goji berries from the Ningxia region in China and freeze-dried samples from Poland. The results showed that naturally dried fruits from the Ningxia region had the highest content of polyphenols and high antioxidant potential. The lowest amount of bioactive compounds was determined in the freeze-dried goji berries cultivated, processed, and packaged in Poland. Lu et al. [31] analyzed *L. barbarum* fruit samples from 13 different regions of China, with their total polyphenol content within a small range of values from 6.89 to 8.25 mg GAE/g DW. According to the TPC values, the fruit of *L. barbarum* produced in Guyuan in Ningxia had the highest value compared to the best-evaluated product from Zhongwei in Ningxia, China. Similarly, Ma et al. [32] evaluated and compared the TPC in goji berries from various regions of China. They found that the total polyphenol content of goji berries in the Ningxia region (183.41 μg/g FW) was higher than in the Qinghai and Gansu (156.81 and 111.17 μg/g FW, respectively) regions.

Another important factor to consider is genotype/cultivar. Genotype variance was also proven for three genotypes of *L. barbarum* fruits from Northern Italy by HPLC fingerprinting. Donno et al. [13] examined the range of total polyphenols in samples of genotypes from Northern Italy, which ranged from 255.87 to 281.91 mg GAE/100 g FW. Zhang et al. [33] quantified the levels of predominant polyphenols present in eight native Chinese goji genotypes. The total polyphenol content ranged from 26.9 to 73.4 mg GAE/g FW, with the ‘Baihua’ genotype having the highest content and ‘Heiguo’ the lowest. The ‘Damaye’, ‘Zhongguo’, ‘Ningji’, and ‘Beifang’ genotypes had significantly higher TPC levels than the other tested genotypes, with TPCs higher than 50 mg GAE/g FW.

Mocan et al. [12] compared two cultivars of *L. barbarum* grown in Northwest Romania. The results of total polyphenol content showed that the cultivars provided different TPC values compared to another cultivar (11.6 and 15.7 mg GAE/g DW, respectively). Wojdyło et al. [34] found high amounts of polyphenols, especially in a new Polish genotype. Kosinska-Cagnazzo et al. [35] evaluated the polyphenolic profile of six goji berry cultivars originating from Switzerland. The analysis of polyphenols proved that the cultivar ‘Number One’ had the highest TPC, with phenolic compounds (identified by HPLC-DAD) such as rutin as well as ferulic, chlorogenic, caffeic, and *p*-coumaric acids. The lowest value was found in the ‘Tibet’ cultivar.

Polyphenol content depends on the stage of ripening. Poggioni et al. [36] observed secondary metabolite accumulation in goji fruits (*L. barbarum* L.). Berries from Italy were analyzed according to the time of harvesting during two ripening seasons. The berries harvested in September showed the highest TPC values (3.22 mg GAE/g). The fact that the stage of ripeness is correlated with the content of phenolic compounds was confirmed by principal component analysis in the study by Zhao et al. [37]. They analyzed the phenolic spectrum of four varieties of goji berry, including four stages of ripeness—green skin, yellow-green skin, orange skin, and red skin. The highest concentrations were shown by the phenolics rutin, isoquercitrin, and chlorogenic acid, with a tendency to decrease during fruit ripening. Caffeic acid was determined only in earlier stages and not at the ripening stage, while *p*-coumaric acid content showed an increase in the first stages followed by a decrease. The mentioned changes in polyphenolic compounds can be explained by increased phenol oxidase activity, a reduction in primary metabolism during fruit ripening stages, and the conversion of phenolic compounds (polymerization, oxidation, and conjugation reactions) [38].

The total polyphenol content is influenced by the extraction method, as confirmed by Stanoeva et al. [39]. Methanol with ascorbic acid (2%) was identified as the most efficient solvent for obtaining the highest TPC values. Methanol solution was more efficient for extraction compared to distilled water or acetone. Aqueous solutions of citric and ascorbic acid yielded highest content of phenolic acids and flavonoids. Benchennouf et al. [16] tested the effect of the extraction method on the TPC in *Lycium barbarum* berries cultivated in Crete. The total phenolic content ranged from 14.13 mg GAE/g DW (water fraction, the least effective) to 109.72 mg GAE/g DW (ethyl acetate fraction, the most effective). The research group of Skenderidis et al. [40] also determined the TPC values of Greek goji fruit (*L. barbarum*) extracts extracted with different solvents (distilled water; a mixture of ethanol and distilled water (70:30); a mixture of hexane, distilled water, and ethanol (33:33:33)). The highest yield of total polyphenols was obtained from the sample extracted with ethanol and distilled water (79.458 mg GAE/g of encapsulated extract dry weight), followed by the sample extracted with water solvent, and the minimum amount of polyphenols (25.307 mg/g) was found in the mixture of hexane, water, and ethanol, as hexane diminished the extraction efficiency.

The different forms of various products prepared with goji berries may affect the final amount of these components. Shang et al. [41] analyzed the phenolic compounds in dried *Lycium barbarum* and a by-product of *L. barbarum* seed oil, isolated by supercritical fluid extraction. They identified 40 phenolic compounds by HPLC-MS/MS, classified as phenolic acids and their derivatives, flavonoids and their derivatives, lignanamides, and other phenolic substances. They detected differences in different goji samples extracted by different methods (the ultrasonic method, reflux method, and enzymatic method). It was noticed that the dried product had higher phenolic content than the by-product of *L. barbarum*. Gallic acid, *p*-coumaric acid, ferulic acid, and chlorogenic acid, from the phenolic acid group, were found in both products. Kaempferol-3-*O*-rutinoside, kaempferol, rutin, rutin hexose, and quercetin were significant flavonoid representatives.

Boleslawska et al. [42] detected different total polyphenol contents in various goji berry products (dried berries, juices, and capsules containing goji berry extract of *L. barbarum*). The results showed that the dried goji berries had the highest content of polyphenols and flavonoids, with the highest AA among all the analyzed products. Significantly lower TPC values were found in the juices, followed by the capsules. Taneva et al. [43] confirmed that dairy products enriched with goji berries displayed higher contents of polyphenols and antioxidant activity compared to control samples. Yogurts with the addition of dried berries had higher phenolic contents and higher AA than control yogurt samples.

To sum up, the TPC depends on the species of goji, cultivar/genotype, extraction and detection method, place of origin of the samples, stage of ripeness, climatic conditions, and the form in which the goji was examined—fresh, dried, or as products.

### 2.1. Polyphenolic Spectrum of Goji Berries

Polyphenolic compounds present in goji berries include flavonoids such as anthocyanins, flavonols, flavones, flavanols, flavanones, as well as isoflavonoids, phenolic acids, and tannins [44,45].

In the study by Mocan et al. [12], representatives of phenolic substances, rutin and ferulic acid, were the most dominant in all goji berry extracts from two Romanian cultivars (Erma and Big Lifeberry), with contents ranging from 8 to 58 μg/g DW and 12.5–55 μg/g DW, respectively.

In goji fruit from a Greek organic farm, seventeen phenolic compounds were identified by Benchennouf et al. [16]. The spectrum included cinnamoylquinic acids and their derivatives, hydrocinnamic acids and flavonoid derivatives, quercetin 3-*O*-hexose coumaric ester, and quercetin 3-*O*-hexose-*O*-hexose-*O*-rhamnose, which were reported for the first time. The most common flavonoids present in berries in Zhong et al.’s [46] study were quercetin-3-*O*-rutinoside and kaempferol-3-*O*-rutinoside, and among the phenolic acids were chlorogenic acid, caffeic acid, and small amounts of caffeoylquinic acid and *p*-coumaric acid. Zhao et al. [47] determined 11 compounds by UPLC/MS/MS (ultra-performance liquid chromatography). Rutin, isoquercitrin, and chlorogenic acid were identified as the main phenolic components present in goji berries during ripening. Their contents decreased with fruit ripening. Zhang et al. [33] examined eleven phenolic compounds in goji fruits, including six flavonoids (quercetin, myricetin, kaempferol, rutin, quercetin-rhamno-di-hexoside, and quercetin-3-*O*-rutinoside) and five phenolic acids (caffeic acid, *p*-coumaric acid, ferulic acid, vanillic acid, and chlorogenic acid). Forino et al. [48] identified phenolic compounds present in goji berries. In addition to previously identified phenolic compounds, including caffeic acid, *p*-coumaric acid, rutin, and scopoletin, they also examined the presence of N-*trans*-feruloyltyramine, N-*cis*-feruloyltyramine, and N-feruloyltyramine dimers.

In another study, phenolic compounds isolated from methanol extracts of goji fruits from Turkey were estimated by LC-MS/MS. The analysis identified flavonoids, phenolic acids, and anthocyanins as the main compounds, especially cyanidin-3-*O*-glucoside, cyanidin chloride, pelargonin chloride (a derivative of cinnamic acid), pelargonidin chloride, and pelargonidin-3-*O*-glucoside. Pelargonidin-3-*O*-glucoside and cyanidin-3-*O*-glucoside were the main anthocyanins in the aqueous extract, with concentrations ranging from 119.60 to 1112.25 mg/kg FW [49]. Covaci et al. [10] analyzed the polyphenolic profile of Macedonian goji berry (*Lycium barbarum* and *Lycium chinense*), finding a predominance of anthocyanins, especially delphinidin-3-*O*-rutoside (880 mg/kg), while salicylic acid predominated among phenolic acids (875 mg/kg). They also identified ellagic acid, ferulic acid, and cinnamic acid (14, 9, and 28 mg/kg, respectively).

Phenylpropanoids represent bioactive compounds with high antioxidant activity. The determination of phenylpropanoids in different medicinal Chinese herbs has confirmed the occurrence of these constituents in goji berry in the amount of 22.7 mg GAE/g [50].

Based on the above findings, polyphenols could be used as biomarkers to distinguish different *Lycium* species or the same *Lycium* genotype from different geographical areas [13].

#### 2.1.1. Phenolic Acids of Goji Berries

Phenolic acids, occurring as abundant compounds, have been evaluated in many research studies. Montesano et al. [51] evaluated goji fruit samples (*L. barbarum)* cultivated in Southern Italy, and identified phenolic acids as the most important phenolic compounds, followed by flavonols and flavanols. Analysis by Donno et al. [13] confirmed this finding in their study of polyphenol groups in goji berries. The most important classes observed were cinnamic acids (7.94%) and catechins (5.99%), followed by flavonols and benzoic acids. Wang et al. [52] identified *p*-coumaric acid as the predominant and most abundant phenolic acid in goji berries, with a content of 6.06 μg/g. According to Wojdyło et al. [34], the content of phenolic acids in 21 new cultivars of goji berries from a breeding program in Poland ranged from 38.91 to 455.57 mg/kg, with an average value of 239.72 mg/kg. The lowest levels of phenolic acids (below 100 mg/kg) were found in 3 out of 21 genotypes. The highest content of phenolic acids was determined for four acids, namely chlorogenic, caffeic, ferulic, and coumaric acids, present in goji fruit in amounts ranging between 111 and 126 mg/100 g FW. Vulić et al. [53] studied goji fruit extract samples cultivated in Serbia. The phenolic acid spectrum analyzed by HPLC included gallic, protocatechuic, vanillic, chlorogenic, coumaric, caffeic, and ferulic acids, with gallic acid predominating (40.44 mg/g). Zhang et al. [33] determined chlorogenic acid to be the most abundant phenolic acid among eight Chinese cultivars of goji, with amounts ranging from 113 to 526 μg/g. Inbaraj et al. [54] quantified 15 phenolic acids in *Lycium barbarum* using the HPLC-DAD-ESI-MS method, with *p*-coumaric acid predominating (64.0 μg/g), followed by caffeic acid (23.7 μg/g) and vanillic acid (22.8 μg/g). Wang et al. [52] and Zhong et al. [46] described chlorogenic and caffeic acids as the predominant phenolic acids. Only trace amounts of caffeoylquinic acid and *p*-coumaric acid were detected by these research groups. Qian et al. [55] mainly identified protocatechuic acid and chlorogenic acid. Donno et al. [13] isolated cinnamic acids, specifically caffeic acid, chlorogenic acid, coumaric acid, ferulic acid (110.84, 113.18, 111.32, and 125.80 mg/100 g DW, respectively), and gallic acid (15.31 mg/100 g DW), as the most dominant fractions of *L. barbarum* berries.

Ozkan et al. [49] evaluated the content of phenolic acids in methanol and water extracts of goji berries (*L. barbarum*) and detected fumaric acid (338.00 and 126.72 mg/kg, respectively), *p*-coumaric acid (77.81 mg/kg and Nd (not detected), respectively), gallic acid (2.95 mg/kg and Nd, respectively), and chlorogenic acid (60.72 and 55.56 mg/kg, respectively). Wojdyło et al. [34] also described the presence of 5-caffeoylquinic acid (89.4%) and 1,5-di-*O*-caffeoylquinic acid (10.6%) in goji berries. Total caffeic acids and their derivates from methanolic extracts of Serbian goji berries represented an amount of 23.91 mg CAE (catechin equivalents)/g, with chlorogenic acid and *p*-coumaric acid present in amounts of 3.39 and 3.32 mg/g DW, respectively [35]. Mocan et al. [12] identified the presence of *p*-coumaroyl-quinic acid isomers of dicaffeoyl-quinic acid, coumaric acid glycoside, and isomers of caffeoyl derivatives in goji berries. Significant phenolic acids included 5-caffeoylquinic acid (89.4%) and 1,5-di-*O*-caffeoylquinic acid (10.6%).

The TPC value in goji berries is cultivar-dependent, as proved by the study by Donno et al. [13]. Three cultivars contained similar amounts of caffeic acid (110.45, 110.44, and 111.65 mg/100 g FW, respectively), chlorogenic acid (114.87, 112.78, and 111.89 mg/100 g FW, respectively), coumaric (111.38, 110.91, and 111.66 mg/100 g, FW respectively)**,** and ferulic acid (125.18, 126.04, and 126.17 mg/100 g FW, respectively).

**Figure 2:**
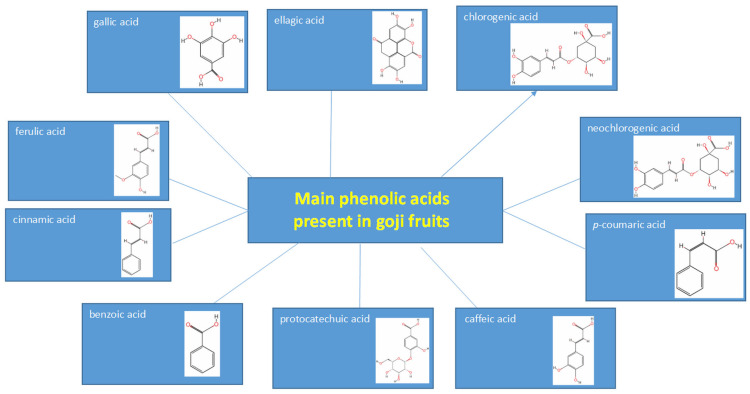
Overview of the most abundant phenolic acids in goji berries.

The research of Gao et al. [56] proved that chlorogenic acid is the major compound responsible for the enzymatic browning of goji berries. Phenolics and flavonoids, whose content first increased and then decreased during storage, correlated with the browning activity. The chlorogenic acid content decreased the fastest under the action of the enzyme, degrading by 75% within 5 min. The amounts of ferulic acid and rutin followed this trend. The most abundant phenolic acids are summarized in Figure 2.

#### 2.1.2. Flavonoids of Goji Berries

Flavonoids, similar to phenolic acids, are important and abundant compounds present in goji berries.

The flavonoid spectrum of goji fruit (*L. barbarum*) was determined by Wang et al. [52], with the most abundant fractions being quercetin-3-*O*-rutinoside, kaempferol-3-*O*-rutinoside, and quercetin-diglycoside. According to Donno et al. [9] and Mocan et al. [12], the predominant flavonol was hyperoside (genotype G, 1115.57 and 2116.90 mg/100 g FW, respectively). Wang et al. [52] detected quercetin-di-glucoside, rutin, and kaempferol-*O*-rutinoside in *L. barbarum* fruits, with rutin being the dominant flavonoid at a concentration of 42.0 μg/g. Inbaraj et al. [54] and Zhang [33] identified quercetin-rhamno-di-hexoside as the dominant flavonoid in native Chinese *L. barbarum* fruits. The rutin content in the fruits of *L. barbarum* cultivated in China was higher (from 0.73 to 1.38 mg/g) than in wild varieties (0.09–0.22 mg/g). Le et al. [57] identified only three flavonoids in *L. barbarum* fruit extract: myricetin, quercetin, and kaempferol. On the other hand, Qian et al. [55] separated flavonoids from *L. chinense* Mill., identifying rutin, hyperoside, hesperidin, morin, and quercetin. Wojdyło et al. [34] identified five flavonols in goji cultivars by LC-MS-Q/TOF (quadrupole time-of-flight): quercetin derivatives (quercetin-di-hexoside, quercetin-rhamno-di-hexoside, quercetin-3-*O*-rutinoside) and two kaempferol derivatives (kaempferol-pentoside-hexoside and kaempferol-3-*O*-rutinoside). Additionally, two aglycones, quercetin-3-glycoside and kaempferol-3-glycoside, were detected. The major compound was quercetin-3-*O*-hexoside (from 169.1 to 1107.7 mg/kg), followed by quercetin-3-*O*-rutinoside (from 7.1 to 232.7 mg/kg). Inbaraj et al. [54] determined by HPLC-DAD-ESI-MS in *L. barbarum* L. 37 flavonoids with a predominance of quercetin, kaempferol, and isorhamnetin derivatives. Quercetin-rhamno-di-hexoside was present in the largest amount (438.6 μg/g), followed by quercetin-3-*O*-rutinoside (281.3 μg/g), dicaffeoylquinic acid isomers (250.1 μg/g), and chlorogenic acid (237.0 μg/g). At lower concentrations were quercetin-di-rhamnohexoside (117.5 μg/g), quercetin-di-rhamno-hexoside (116.8 μg/g), kaempferol-3-*O*-rutinoside (97.7 μg/g), and isorhamnetin-3-O-rutinoside (72.1 μg/g). Among flavonoids in water extract, Ozkan et al. [49] detected quercetin (2.12), isoquercetin (2.90), luteolin-7-*O*-glucoside (8.8), epicatechin (8.63), epigallocatechin (5.37), epigallocatechin gallate (5.23), and kaempferol-3-*O*-rutinoside (13.37 mg/kg DW) in methanol extract.

Zhang et al. [33] examined quercetin-rhamno-di-hexoside and quercetin-3-*O*-rutinoside. Quercetin-rhamno-di-hexoside was identified as the predominant flavonoid. Its content was cultivar-dependent, ranging from 434.7 (Baihua) to 1065 μg/g FW (Damaye). The rest of the analyzed cultivars, such as Ningji No.1, Zhongguo, Yunnan, and Heiguo, had concentrations of quercetin-rhamno-di-hexoside over 800 μg/g FW. Significant differences in quercetin-3-*O*-rutinoside content were also noticed, ranging from 158.9 (Beifang) to 628.9 μg/g FW (Ningji No.1). Other cultivars reached quercetin-rhamno-di-hexoside concentrations of more than 500 μg/g FW. Quercetin represented the third most predominant flavonoid, with concentrations ranging from 89.6 (Ningxiahuangguo) to 369.8 μg/g FW (Baihua), while others contained more than 200 μg/g FW. Myricetin amounts ranged from 16.89 (Baihua) to 117.3 μg/g FW (Zhongguo), and the content of kaempferol varied from 15.3 (Ningxiahuangguo) to 93.6 μg/g FW (Baihua). Rutin had the lowest concentration values among the evaluated flavonoids, with concentrations from 43.2 to 76.1 μg/g FW (Heiguo).

Mocan et al. [12] also detected eriodictyol glucosides such as eriodictyol-3-*O*-glucosyl-*O*″-dihydrocaffeate (C_30_H_29_O_15_^−^), quercetin-3-*O*-caffeoyl-glucoside, and rutin. Donno et al. [13] proved the presence of catechin (118.76) and epicatechin (229.18 mg/100 g FW). The concentration was genotype-dependent for catechins (G1 118.26; G2 118.68; G3 119.35 mg/100 g FW) and epicatechins (G1 227.74; G2 230.54; G3 229.26 mg/100 g FW).

Values of TFC (total flavonoid content) are cultivar-dependent, as shown in the study by Vidovic et al. [1]. They analyzed goji samples from Serbia and identified different values of TFC (0.27–12.32 mg/g DW). According to Ilić et al. [25], they evaluated the TFC of goji berries with a value of 4.76 mg/g DW. Lu et al. [31] analyzed *L. barbarum* fruit samples from 13 different regions in China and found TFC values varying from 0.1812 to 0.4391 mg RE (rutin equivalents)/g DW. Islam et al. [29] provided a comparison of flavonoid amounts in black and red goji fruits. Similar to TPC, higher content of flavonoids was found in black goji berry samples (12.32, 11.90, 10.37, and 9.77 mg CAE/g DW) than in red goji berry samples (2.67, 2.69, 2.78, and 3.16 mg CAE/g DW).

Du et al. [58] described the changes in flavonoid content of goji berry juice during fermentation with Tibetan kefir grains (TKGs). The results showed that TKGs grew well in the juice, and the content of total soluble flavonoids in the juice significantly increased, from 42.28 to 85.69 mg RE/100 mL, after complete fermentation. It was determined that alpha-amylase and xylanase were significantly responsible for the release of flavonoids such as mangiferin, rutin, hyperoside, isoquercitrin, and quercetin during fermentation.

Ma et al. [32] tried to explore the possible molecular mechanism of flavonoid accumulation. According to their findings, the MYB1 gene positively regulates the expression of quercetin-3-*O*-glucoside, quercetin-7-*O*-glucoside, and isohyperoside. Several genes and related transcription factors control the variation in flavone accumulation in goji berries. This can be important for understanding the accumulation and molecular mechanisms of goji flavonoids.

**Figure 3:**
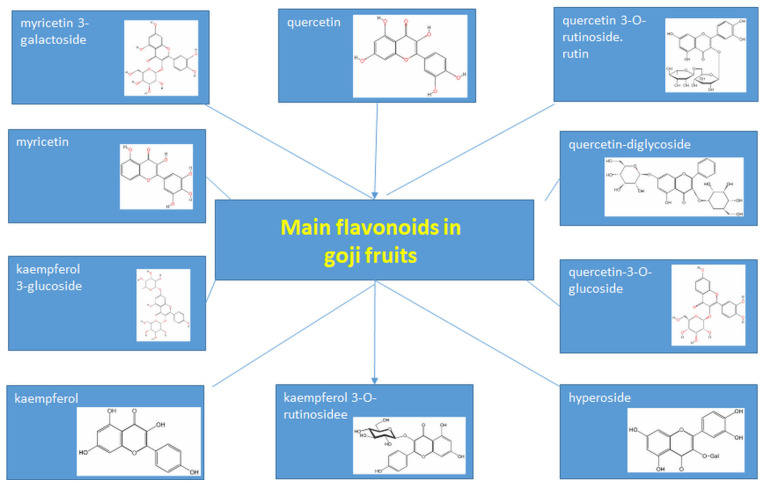
Overview of the most dominant flavonoids in goji fruits.

Differences in polyphenol and flavonoid composition relative to published literature data may be due to differences in *Lycium* species, growth conditions, and different extraction and purification parameters. The most dominant flavonoids can be seen in Figure 3.

#### 2.1.3. Anthocyanins in Goji Berries

Anthocyanins represent the predominant flavonoid compounds in *Lycium ruthenicum* fruit extract, as stated by Sharma et al. [59]. They comprise about 2.3% of the dried fruit, with the highest reported content being 24.04 mg/g [60]. In contrast, anthocyanins have not been investigated at any stage of fruit development in *L. barbarum* [61].

*Lycium ruthenicum* fruits contain over ten times more anthocyanins compared to raspberries and grapes. The compounds occurring in the largest quantities in black goji berries are monoacylated anthocyanins derived from petunidin. Petunidin derivatives constitute more than 95% of the black goji anthocyanin spectrum. They can be characterized by 3,5-diglycosylation and acylation by phenolic acids, such as ferulic, *p*-coumaric, and caffeic acids, and the coexistence of *cis*- and *trans*-isomers [62,63]. Acylated anthocyanins present in berries show better stability than non-acylated ones, as analyzed in berry crops such as blackberries and blueberries [64,65]. Tang et al. [66] examined the anthocyanin profile (HPLC/PDA/MS) of black goji berry extract. They identified petunidin derivatives, with *cis-* and *trans*-isomers of petunidin-3-*p*-coumaroyl-rutinoside-5-glucoside, as predominant. The *trans*-isomer of petunidin derivatives contributed the most to the color expression of black goji extract and showed the highest stability [67]. Acylation enhanced the color retention of petunidin derivatives and increased color intensity and stability. In terms of petunidin concentration in *L. ruthenicum* berry extract, it was 5.81 mg/g DW, with delphinidin being the second most abundant (0.34 mg/g DW) [45].

*Lycium ruthenicum* berries also contain anthocyanins in methanol extract, including pelargonin chloride (3.35 mg/kg DW), pelargonidin chloride (19.52 mg/kg DW), and cyanidin chloride (1.53 mg/kg DW). In water extract, they contain cyanidin-3-*O*-glucoside (1112.25 mg/kg DW) and pelargonidin-3-*O*-glucoside (119.60 mg/kg DW), as demonstrated by Ozkan et al. [49]. Wang et al. [68] also determined anthocyanins in the extract of stored, air-frozen *L. ruthenicum*. They detected anthocyanidins, with delphinidin as the most abundant (about 51%), followed by cyanidin, petunidin, malvidin, and peonidin. Not only do freezing and storage affect the anthocyanin profile, but the stage of maturity and cultivar/genotype of *Lycium ruthenicum* fruit also significantly influence it [69]. Stanoeva et al. [39] proved that the yield of anthocyanins can be significantly influenced by the extraction solvents used for their isolation, with the highest yield obtained using methanol/water/ascorbic acid in a 7:28:2 ratio. Fatchuraman et al. [20] determined anthocyanins based on the storage conditions of goji berries at three different temperatures (0, 5, and 7 °C) for 12 days. Significant changes were observed in anthocyanin content, with a storage temperature of 5 °C for 9 days being most suitable to maintain the higher quality of fresh goji berries.

#### 2.1.4. Antioxidant Activity of Goji Berries

Total antioxidant activity (TAA) can be evaluated by several methods to determine free radical scavenging activity, such as methods using DPPH (2,2′-diphenyl-1-picrylhydrazyl) and ABTS (2,2′-azino-bis (3-ethylbenzothiazoline-6-sulfonic acid)) reagents, and the FRAP (Ferric Reducing Antioxidant Power) assay.

FRAP analysis has been performed to determine the total antioxidant activity of goji fruits from Northern Italy, in comparison with other types of berries and other fruits. The results indicated differences between the fruits. Berries showed the highest antioxidant activity, especially blackcurrant and blueberry (76.86 and 49.36 mmol Fe^2+^/kg, respectively). Goji berries showed a higher FRAP value (19.36 mmol Fe^2+^/kg) than kiwifruit, raspberry, apple, and orange [9]. *Lycium* fruit extracts, evaluated by the DPPH free radical scavenging activity, ABTS, and FRAP methods, showed similar TAA values (49.65 μM TE/g FW) to bayberry fruit extracts (47.8 μM TE/g FW) and blackberry extracts (56.3 μM TE/g FW) [70].

Vulić et al. [53] studied goji fruit extract samples cultivated in Serbia. Antioxidant potential was examined by measuring the ability to scavenge DPPH and hydroxyl radicals using methanol and hexane as solvents. IC_50_ values of the DPPH· method ranged from 26.64 μmol TE/g for hexane extract to 62.15 μmol TE/g for methanol extract. Reducing-power values ranged from 952.23 μmol TE/g for methanol extract to 1360.48 mg/mL for hexane extract. Covaci et al. [10] compared the TAA of *Lycium barbarum* berries cultivated in Macedonia to *L. chinense*. The average TAA values of the *L. barbarum* berry extracts were about half those of the *L. chinense* variety. The antioxidant potential measured by the PCL (photochemiluminescence) method had a TAA of 2037 mg TE/kg. Similarly, Skenderis et al. [27] compared the DPPH and ABTS free radical scavenging activities of *L. barbarum* and *L. chinense* berries. The results confirmed that both assayed species had high antioxidant activity, with the DPPH TAA ranging from 784 to 1254 μg/mL and ABTS values ranging from 192 to 407 μg/mL. Moreover, the antioxidant capacity based on the half-maximal inhibitory concentration (IC_50_) measurements of DPPH and ABTS free radical scavenging showed better values for *L. barbarum* than *L. chinense* berries. Mocan et al. [12] also examined both goji berries by the DPPH radical scavenging assay, acquiring similar results: 8.79 and 9.35 mg TE/g. Ağagündüz et al. [24] determined the antioxidant activity of dry goji berry fruit (*Lycium barbarum*) from Turkey by the ferric reducing ability of plasma (FRAP) assay. Their analyses determined the antioxidant activity at 32.6 μmol TE/g. Benchennouf et al. [16] tested the TAA of *Lycium barbarum* berries cultivated in Crete after degreasing and extraction with dichloromethane and methanol using a Soxhlet apparatus, and fractionation with ethyl acetate and butanol solvents. The ethyl acetate extract had the highest scavenging activities (as IC_50_) using DPPH and chemiluminescence assays (4.73 and 0.47 mg/mL, respectively).

Ilić et al. [25] evaluated the TAA of *L. ruthenicum* fruit, originating from Serbia, by the DPPH method (58.65 μM TE/g) and ABTS (144.7 μM TE/g) and FRAP assays (61.75 μM TE/g). The TAA values of *L. ruthenicum* fruit extract were stated to be higher than those of other *Lycium* species [11]. Wojdyło et al. [34] examined the antioxidant activity of 21 new cultivars of goji (*Lycium barbarum* L.) using the ABTS and FRAP methods. The results of the ABTS and FRAP assays varied from 1.60 to 6.83 mmol TE/100 g and from 1.44 to 6.30 mmol TE/100 g, respectively. The authors also identified the most promising cultivars. Zhang et al. [33] analyzed eight samples of Ningxia goji (*Lycium barbarum* L.) genotypes. The Ningxiahuangguo genotype was rich in polyphenols and showed significantly higher TAA values than those of the other genotypes. Differences among analyzed genotypes were also demonstrated by the study of Donno et al. [13].

Islam et al. [29] compared the DPPH and ABTS radical scavenging activity of eight black and red goji berry samples. The measurements proved that higher values of DPPH and ABTS activities were detected in black goji berry samples (35.86, 35.68, 33.30, and 32.90 µmol TE/g for DPPH and 180.03, 167.59, 150.51, and 147.00 µmol TE/g for ABTS) in comparison to red goji berry samples (16.07, 16.46, 16.61, and 17.47 µmol TE/g for DPPH and 53.92, 55.87, 62.40, and 64.38 µmol TE/g for ABTS).

The most important anthocyanin in *L. ruthenicum* fruit, identified by Tang et al. [71] as petunidin 3-*O*-[6-*O*-(4-*O*-(*trans*-*p*-coumaroyl)-α-l-rhamnopyranosyl)-β-d-glucopyranoside]-5-*O*-[β-d-glucopyranoside], exhibited excellent antioxidant activity in vitro and had better AA than a crude extract of anthocyanins determined by DPPH, superoxide, and ABTS assays.

It is important to observe TAA and its changes in goji berry products that are prepared from the fruits. Liu et al. [72] studied goji berry infusion, a traditional herbal drink prepared from red and black goji berries. The research studied the effect of water temperature and soak time on the antioxidant potential (ABTS, DPPH, and FRAP assays) of two goji berry drinks. They stated that both red and black goji berry drinks have a high amount of bioactive compounds that contribute to high antioxidant potential. The levels of bioactive compounds and the antioxidant activity of both the goji berry drinks increased with increasing soak temperature and time. The black goji berry drink displayed higher values of TAA in comparison to the red one. Preparing the drink with 100 °C water increased the TAA value (550 μmol/100 mL) of the black goji berry drink by five times compared to the red goji berry drink.

Liu et al. [73] prepared fermented goji berry juices from *Lycium barbarum* L., containing bacterial strains obtained from fermented foods, *Bacillus velezensis*, *Bacillus licheniformis*, and *Lactobacillus reuteri* mixed with *Lactobacillus rhamnosus* and *Lactobacillus plantarum.* They tested the antioxidant activity of these juices attributed by the used microorganisms. The TAA was improved significantly in the fermented juices as the fermentation influenced the conversion of the free and bound forms of phenolic acids and flavonoids in the juices. The TAA values were correlated with the phenolic composition.

Goji fruit antioxidant activity is affected by several significant compounds or groups of components, such as polyphenolic compounds, phenolic acids, flavonoids such as anthocyanins, and others. Liu et al. [71] reported that phenolics were the most significant for the antioxidant potential of *Lycium ruthenicum* fruit extract, especially considering the anthocyanin content. The positive linear correlation between phenolic compounds and antioxidant activities in goji berries was also exhibited by Islam et al. [29]. TAA was positively correlated with the content of carotenoids, flavonoids, and polysaccharides, as evaluated by Guo et al.; Jiang et al.; Lin et al.; and Wang et al. [31,52,74,75]. Phenolic compounds, especially flavonoids, phenolic acids, and condensed tannins, contribute the most to the antioxidant capacities of goji fruit, as discovered by Cai et al. [76]. Antioxidant activity shows a strong positive correlation with total polyphenols (r = 0.9996), as Donno et al. [13] stated. According to Wang et al. [52], flavonoids in goji berries exhibited the strongest effect in removing free radicals. The correlation between TAA values, measured by the ABTS or FRAP method, and total polyphenolic content reached up to r = 0.523 and 0.038, respectively; for phenolic acids, r = 0.277 and r = 0.328, respectively; and for flavonols, r = 0.531 and r = 0.506, respectively, as published by Wojdyło et al. [34].

## 3. Health-Promoting Activity of Goji Berries

Considering the many published studies, it can be said that goji berries display numerous health-promoting activities [3,76,77,78]. Goji berries can be utilized in the prophylaxis of several diseases, such as diabetes, cardiovascular diseases, and cancer. However, attention should also be focused on people who suffer from food allergies and intolerances due to the high degree of cross-reactivity between goji and several types of fruits such as peach and tomato [3].

It is known that the presence of polysaccharides, glycopeptides, polyphenols, flavonoids, carotenoids, and their derivatives is related to the health potential of goji berries [78]. Despite the fact that the majority of studies have focused on polysaccharides and carotenoids in goji fruits and their benefits, goji’s phenolic compounds also exert strong effects such as antioxidant and anti-inflammatory. Polyphenols from goji berries can be used to treat different diseases influenced by oxidative stress and inflammation, which are some of the causes of diabetes, neurological and cardiovascular diseases, and cancer [25]. Phenolic compounds in goji fruits such as rutin, isoquercitrin, and chlorogenic acid play an important role in the prevention of metabolic and chronic diseases [27,79]. Many animal research studies and in vitro cell culture studies have shown a significant effect of the application of *Lycium barbarum* berries, with the predominant antioxidants being polyphenols and carotenoids, on protection against different peroxidation-related conditions such as lipid peroxidation [80,81,82,83,84].

The study by Lee et al. [85] confirmed that most of the health properties of *Lycium ruthenicum* are related to the presence and synergism of flavonoids such as anthocyanins and functional polysaccharides. Phenolics of black goji (*Lycium ruthenicum*) berries, like anthocyanins, show positive health benefits such as antioxidant, anti-inflammatory, hypoglycemic, hypolipidemic, and antimicrobial properties [86,87]. The study by Ilić et al. [88] proved that the flavonoid extract of *Lycium ruthenicum* also has prebiotic properties.

### 3.1. Neuroprotective Effect of Goji Berries

**Figure 4:**
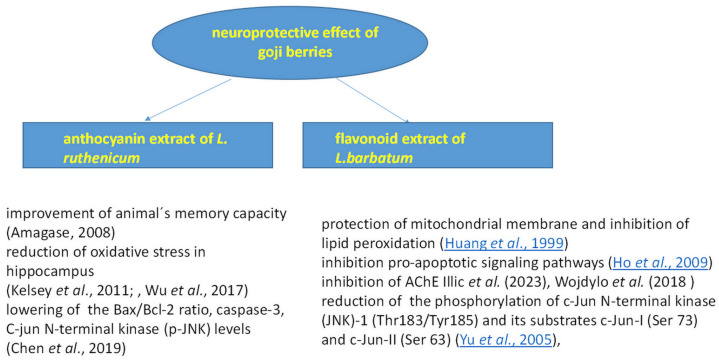
Neuroprotective effect of goji berries [37,44,81,83,90,91,94,95,96].

Free radicals, as products of oxidation, play an important role in many diseases and age-related problems. High antioxidant potential is related to the presence of bioactive compounds, polyphenolic compounds that, together with polysaccharides in goji berries, can be utilized in the future as alternative therapeutic agents for neurodegenerative diseases such as Alzheimer’s and others [89], as shown in Figure 4.

A positive effect of *Lycium ruthenicum* Murr. anthocyanin extract was shown in an animal model of amyloid beta-protein 1–42-induced Alzheimer’s disease. An extract concentration of 80 mg/kg significantly improved the animal’s memory capacity and significantly reduced markers of neuronal oxidative stress and inflammation in the hippocampus tissue of a rat brain [90,91].

Bioactive compounds in goji berries display antioxidant potential that is important for the prevention of brain oxidative mitochondrial damage. They are also significant in the prevention of cognitive dysfunction associated with prenatal stress, as reported by Haiyang et al. [92]. An extract isolated from *L. barbarum* berries, containing flavonoids, displayed a strong antioxidant effect, and mitochondrial lipid peroxidation was significantly inhibited by flavonoids from *L. barbarum* in a dose-dependent manner, as published by Huang et al. [93]. In another study, the scavenging effects of goji berries were proven using the ESR–spin trapping technique. Flavonoids scavenged free radicals in the xanthine/xanthine oxidase (Xan/XO) system, and in the Fenton reaction [93].

The neuroprotective effect of *L. barbarum* fruit can be explored not only through antioxidant action but also by inhibiting pro-apoptotic signaling pathways. Goji berries also showed a neuroprotective effect against homocysteine [94].

Another treatment option for neurodegenerative disorders is the inhibition of acetylcholinesterase (AChE), responsible for the breakdown of the acetylcholine neurotransmitter. Berries of *L. barbarum* inhibited AChE, as evaluated by Mocan et al. [12]. Illic et al. [88] observed the inhibition of AChE by *Lycium ruthenicum* flavonoid extract. However, the inhibition rate of *L. ruthenicum* extract was much lower than that of *L. barbarum*. This observation can be explained by the higher content of zeaxanthin in *L. barbarum*. Wojdyło et al. [34] compared the inhibitory activity of 21 new goji cultivars. The results showed that goji fruits are effective in inhibiting AChE by 50% (from 29.9 to 46.3%) and also BuChE (butyrylcholinesterase) by 30% (from 4.9 to 27.0%). The inhibitory activity significantly depends on the genotype.

Pre-treatment with aqueous *L. barbarum* fruit extract lowered the release of lactate dehydrogenase (LDH) activity. Yu et al. [95] investigated the mechanisms of the neuroprotective effects and confirmed that the prepared fruit extract reduced the phosphorylation of JNK-1 (Thr183/Tyr185) and its substrates c-Jun-I (Ser 73) and c-Jun-II (Ser 63).

The neuroprotective effect of *Lycium ruthenicum* berry extract with anthocyanins on D-galactose-treated rats was studied by Chen et al. [96]. Ethanol extract from dried goji fruits with a high content of anthocyanins reduced RAGE (receptor for advanced glycation end products) and inhibited oxidative stress caused by D-galactose. Anthocyanins reduced the overexpression of NF-κB (nuclear factor kappa B), IL-1β (inter-leukin-1-β), and p-JNK (C-jun N-terminal kinase), thus inhibiting memory dysfunction and neuroinflammation, possibly through the RAGE/NF-κB/JNK pathway.

Hu et al. [97] studied the neuroprotective effect of polyphenolic glycosides such as lyciumserin A (C_36_H_42_O_21_) and lyciumserin B (C_42_H_52_O_26_), isolated from *Lycium ruthenicum* extracts. They showed the moderate inhibitory activity of monoamine oxidase B (MAO-B), finding MAO-B inhibition rates of more than 50% at a concentration of 100 μM. They demonstrated important neuroprotective properties (69.22 and 72.38% cell viability, respectively) in the 6-hydroxydopamine-induced injury of the PC12 cell model (54.41%).

### 3.2. Metabolic, Anti-Obesogenic, and Antidiabetic Effect of Goji Berries

**Figure 5:**
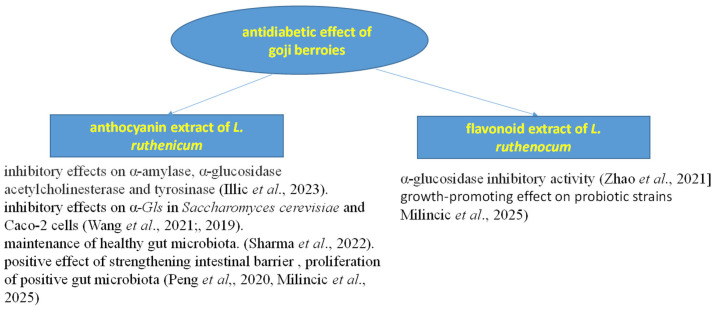
Antidiabetic effect of goji berries [44,46,59,68,100,106,107].

Goji berries represent a significant source of polysaccharides, carotenoids, fiber, phenolic compounds, vitamins, and minerals, which display pro-metabolic effects [98]. Rutin, isoquercitrin, and chlorogenic acid, significant phenolics in goji berries, directly provide antioxidant, hypoglycemic, and hypolipidemic effects, further enhancing the berries’ potential to support metabolic health and decrease the occurrence of chronic diseases [27]. Goji berries improve lipid and glucose metabolism (Figure 5), reduce oxidative stress, and indirectly affect human health by regulating gut microbiota [79]. Three weeks of regular consumption led to a decrease in triglyceride levels, increased HDL cholesterol levels (by about 10–15 mg/dL), and reduced fasting glucose concentrations (by about 7–6 mg/dL), as detected by Antonelli et al. [79]. Krucek et al. [99] also proved the presence of in vitro antidiabetic properties of fruit extracts isolated from *L. barbarum* and *L. chinense*. A study by Wojdyło et al. [34] proved in vitro antidiabetic potential too (the inhibition of alpha-amylase and alpha-glucosidase) in a Polish goji genotype. *Lycium ruthenicum* anthocyanin extract demonstrated in vitro inhibitory effects on α-amylase (6.56 mg/mL), α-glucosidase (7.28 mg/mL), acetylcholinesterase (5.28 mg/mL), and tyrosinase (5.32 mg/mL). Inhibition of these enzymes may reduce the increase in blood glucose levels, leading to the suppression of postprandial hyperglycemia, essential for the management of type 2 diabetes mellitus [88].

Goji anthocyanins display inhibitory effects on α*-Gls* in *Saccharomyces cerevisiae* and Caco-2 cells. The IC_50_ values for these inhibitory effects were 1.32–1.57 μg/mL and 25.3 μg/mL, respectively, comparable to acarbose, a known α-Gl inhibitor [68,100]. Similarly, the phenylpropanoid derivative ethyl-*p*-*trans*-coumarate isolated from *Lycium ruthenicum* flavonoid extract demonstrated α-glucosidase- and α-Gl-inhibitory activity, similar to the positive control (acarbose) [37].

Anthocyanins, flavonoids isolated from *L. ruthenicum* extract, were determined to have an anti-obesogenic effect on metabolism. They lower body weight in animal models by changing the gut microbiota [101,102]. A study by Yin et al. [103] proved that the consumption of an anthocyanin-rich extract of *L. ruthenicum* Murr. was significant for a reduction in body weight and decrease in the Lee index.

According to Sun et al. [104], the regular consumption of goji berries decreased the prevalence of inflammatory bowel disease (IBD). A wide range of bioactive compounds such as polysaccharides, carotenoids, and polyphenols contribute to prebiotic effects, which can prevent dysbiosis associated with IBD. Several studies have shown that anthocyanin metabolites have probiotic activities, maintaining healthy gut microflora. They significantly increase the occurrence of *Bifidobacterium* [59]. Skenderidis et al. [105] examined the prebiotic properties of goji berry powder on selected probiotic bacteria grown in a nutritive synthetic substrate and in simulated gastric and intestinal juices. The selected probiotic strains of *Bifidobacterium* and *Lactobacillus* were cultivated in these substrates with/without the addition of encapsulated goji berry extracts with various contents of polysaccharides and polyphenols. The addition of the goji berry powder increased the proliferation of probiotic strains. According to the work of Peng et al. [106], the prebiotic effect is positively correlated with goji berry polysaccharides and/or polyphenols. They showed that the application of crude extract of anthocyanins from *L. ruthenicum* Murr. fruits to mice had a positive effect on strengthening the intestinal barrier (through the regulation of several barrier proteins) and the proliferation of positive intestinal microbiota (*Coprobacter*, *Barnesiella*, *Eisenbergiella*, *Alistipes*, and *Odoribacter*). A study by Milinčić et al. [107] proved that lyophilized fruit extracts of *Lycium ruthenicum* Murr. and *Lycium barbarum* L. have a growth-promoting effect on probiotic strains in a dose-dependent manner (0.3–5 mg/mL). Therefore, they recommended using these extracts, containing anthocyanins and some phenylamides, as supplements or functional food additives.

Lu et al. [108] stated that *Lycium ruthenicum* fruit extract has a positive effect on liver health problems, inhibiting the development of non-alcoholic fatty liver disease in mice, caused by cholesterol and a high-fat diet. Serum aspartate transaminase (AST) levels were significantly increased in the group fed a Western diet (WD) compared to the group fed a normal diet. AST levels and the size of hepatic fat droplets were reduced in the WD group supplemented with *L. ruthenicum* fruit extract. Also, fatty acid oxidation was increased, and fatty acid synthesis in the liver reduced, leading to an amelioration of the development of non-alcoholic fatty liver disease induced by WD. Chen et al. [109] proved the alleviating effect of ethanolic extract of dried *Lycium ruthenicum* anthocyanin supplementation on D-galactose-induced liver damage. The D-galactose-treated group displayed an unclear structure of hepatocytes and cell necrosis, which was improved by goji anthocyanin treatment. The intake of goji anthocyanins reversed the results by reducing D-galactose-induced serum AST, alanine transaminase (ALT) levels, and blood LDH concentrations. AST and ALT levels correspond to the extent of liver damage. LDH concentrations correspond to the extent of cellular damage and inflammation. Goji anthocyanin treatment mitigated D-galactose-induced hepatocyte death by reducing this mRNA level.

### 3.3. Anti-Inflammatory and Antimicrobial Effect of Goji Berries

**Figure 6:**
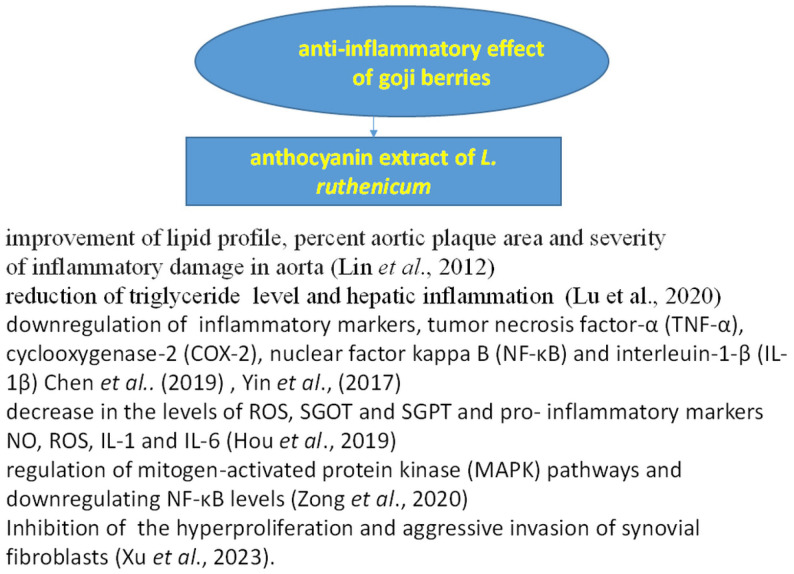
Anti-inflammatory effect of goji berries [4,96,103,111,112,113,114].

Generally, polyphenols and their metabolites are able to reduce inflammation and modulate the intestinal microbiota [88,110], as shown in Figure 6.

The anti-inflammatory effect of anthocyanins, isolated from *L. ruthenicum* Murr. berries (doses of 100, 200, and 400 mg/kg, fed for 6 weeks), on a hyperlipidemia atherosclerosis animal model (mice) was studied by Lin et al. [111]. They stated that anthocyanins significantly improved the severity of inflammatory damage in the aorta, heart, and liver tissue in comparison to the simvastatin drug group. The administration of anthocyanin-rich *L. ruthenicum* extract brought positive results in a pre-clinical animal model of Western diet-induced fatty liver disease. The animal group that consumed the fruit extract displayed reduced hepatic inflammation [108]. Hou et al. [112] studied the anti-inflammatory effect of anthocyanin extract of *L. ruthenicum* Murr. in a rat model of carbon tetrachloride-induced hepatic injury. The results showed that oral administration of *L. ruthenicum* extract (30 mg/kg) for 7 days caused a significant decrease in the levels of pro-inflammatory markers NO, ROS, IL-1, and IL-6. Moreover, Chen et al. [109] showed the mechanism of action of anthocyanins in the regulation of inflammatory markers, tumor necrosis factor-α (TNF-α), cyclooxygenase-2 (COX-2), nuclear factor kappa B (NF-κB), and interleuin-1-β (IL-1β), which are hallmarks of inflammatory diseases. Another possibility to decrease colon inflammation is by regulating mitogen-activated protein kinase (MAPK) pathways and the reduction in NF-κB levels [113]. Yin et al. [103] showed that the consumption of anthocyanin-rich extract of *L. ruthenicum* Murr. is important for the regulation of the established pro-inflammatory markers TNF-α, IL-6, NF-κB, IFN-γ, and iNOS.

Another practical application of anthocyanin extract of *Lycium ruthenicum* is the treatment of rheumatoid arthritis. Goji anthocyanins (100, 200, and 400 μg/mL) notably inhibited the hyperproliferation and aggressive invasion of synovial fibroblasts and reduced cell viability in a concentration-dependent manner. Immunosuppressive reactions, a major side effect of methotrexate, a conventional chemotherapeutic agent, were not observed with goji anthocyanins [114].

Phenolic extracts of goji berries display antimicrobial effects against some Gram-negative and Gram-positive bacteria, as presented by Pires et al., Ilic et al. [11,115], and Shah et al. [116]. A preliminary in vitro study with *L. barbarum* extract showed the significant antibacterial effects of goji berry extract on 17 kinds of bacteria, including *Staphylococcus aureus*, *Staphylococcus epidermidis*, *Salmonella* Typhi, *Salmonella* Paratyphi, *Salmonella* Typhimurium, *Bacillus subtilis*, *Bacillus anthracis*, *Pseudomonas aeruginosa*, *Bacillus dysenteriae* (*Shigella dysenteriae*), *E. coli*, *Candida albicans*, and *Typhoid bacillus* [117]. On the other hand, the study by Ilic et al. [11] showed that methanol extract from black goji berry (0.125–2 mg/mL) did not inhibit the growth of Gram-positive and Gram-negative bacteria and the yeast *Candida albicans*. The best antimicrobial activity was obtained with yellow goji berry extract, which inhibited the growth of three Gram-negative strains (*K. pneumoniae*, *S. abony*, and *P. aeruginosa*) and yeast *C. albicans* at 2 mg/mL. This can be explained by the higher concentration of flavonoids in the yellow goji berry extract compared to other analyzed extracts. Skenderidis et al. [27] evaluated the antimicrobial properties of water and ethanol ultrasound-assisted extracts of dry goji berries. They determined the antimicrobial activity against several species of foodborne Gram-negative bacteria (*Escherichia coli*, *Salmonella typhimurium*, and *Campylobacter jejuni*), Gram-positive bacteria (*Staphylococcus aureus*, *Listeria monocytogenes*, and *Clostridium perfringens*), yeasts (*Yarrowia lipolytica*, *Metschnikowia fructicola*, and *Rhodotorula mucilaginosa*), and fungi (*Penicillium expansum*, *Aspergillus niger*, *Fusarium oxysporum*, and *Rhizoctonia solani*). Although the goji berry samples displayed strong antibacterial activity, they had minimal or no antifungal effect. Antimicrobial properties seem to be related to the polyphenol content.

In the study by Ilić et al. [88], they proved that *Lycium ruthenicum* extract most stimulates the growth of the yeast *Saccharomyces boulardii* among all the tested microorganisms, 2.8 times more than the extract of *L. barbarum*. The important prebiotic properties could be affected by the significant anthocyanin amount that is the major part of *L. ruthenicum* extract. Peng et al. [118] found the in vitro conditions needed for the modulatory properties of *Lycium ruthenicum* anthocyanins and their metabolites on probiotic (*Lactobacillus* and *Bifidobacterium)* and pathogenic bacteria (*Escherichia/Shigella* species). One of the possible mechanisms of action is the selective fermentation of anthocyanins to some short-chain fatty acids.

### 3.4. Anticancer Activity

Several studies have determined that compounds present in goji berries exhibit pro-apoptotic and antiproliferative activities against cancer cells [119].

Miranda et al. [78] examined several fractions extracted from goji berries that demonstrated antitumor properties and were efficient against breast cancer without showing cytotoxic effects on normal human cells. They studied the biochemical mechanisms modulated in breast cancer cells and investigated the antitumoral properties of ethanolic extract of *Lycium barbarum* fruits on different breast cancer cell lines. The extract showed activation of the P-IRE1 alpha/XBP1/NLRP3 axis in MCF-7 cells. It did not exhibit cytotoxicity against healthy human cells but manifested antioxidant qualities by neutralizing ROS (reactive oxygen species) generated by doxorubicin. These results highlight the potential of goji berries as a promising natural product in cancer treatment.

To sum up, further studies are needed to explore and understand deeper the potential mechanism of action of polyphenols, the biological activity of polyphenol metabolites, and their interaction with the microbiome in goji berries. Moreover, no studies have focused on the bioaccessibility and bioavailability of polyphenols in goji berries. Studies on bioaccessibility have mainly focused on carotenoids in goji berry (*Lycium barbarum* L.) using in vitro digestion models and metabolomics approaches [120].

Members of the Solanaceae plant family, including the goji plant, contain alkaloids such as tropane (L-hyoscyamine, scopolamine) and steroidal alkaloids (alpha-solanine, alpha-chaconine), whose content decreases with the ripeness of goji fruit [121]. Magnetic fields can also be used to reduce the quantities of toxic glycoalkaloids during storage, thereby improving their postharvest quality [122].

## 4. Conclusions

Goji berries of *Lycium barbarum*, *Lycium chinense,* and *Lycium ruthenicum* are reported to be rich sources of bioactive substances. These compounds mainly belong to the group of polyphenols, with the main representatives being phenolic acids and flavonoids. Other nutrients and biologically active compounds include lipids, polysaccharides, carotenoids, alkaloids, sterols, tocopherols, and terpenoids. All representatives of *Lycium* spp. are known for their health benefits related to the presence of polyphenols, polysaccharides, and carotenoids. The biological qualities of goji berries include neuroprotective and anti-inflammatory effects. They play an essential role in the prevention of diabetes, cardiovascular diseases, and cancer. Their cytotoxic effect against some cancer cell lines is mainly attributed to flavonoids, especially anthocyanins, isolated from *L. ruthenicum* berries.

## Data Availability

No new data were created or analyzed in this study. Data sharing is not applicable to this article.
